# ReaGP: integrating residual units and attention mechanisms in convolution neural network for genomic prediction

**DOI:** 10.1186/s12711-025-01015-8

**Published:** 2026-01-13

**Authors:** Jing Li, Peng Guo, Yuanxu Zhang, Haoran Ma, Zhida zhao, Yuanqing Wang, Zezhao Wang, Yan Chen, Lingyang Xu, Lupei Zhang, Huijiang Gao, Xue Gao, Junya Li, Bo Zhu

**Affiliations:** 1 Institute of Animal Science, Chinese Academy of Agricultural Sciences, Beijing, 100193 China; 2 College of Computer and Information Engineering, Tianjin Agricultural University, Tianjin, 300384 China; 3 National Centre of Beef Cattle Genetic Evaluation, Beijing, 100193 China; 4 Northern Agriculture and Livestock Husbandry Technology Innovation Center, Hohhot, 010010 China

## Abstract

**Background:**

Various methods have been widely utilized to estimate the genomic breeding values (GEBVs) for genomic prediction. Traditional approaches often relied on the assumption of linear regression models, which struggle to effectively capture the nonlinear relationships between limited phenotypic data and high-dimensional genotypic data. Deep learning (DL) provided a powerful solution for addressing nonlinear problems. Herein, we proposed a novel deep learning method, named residual attention genomic prediction (ReaGP), which was characterized by two main features. It employed residual units to mitigate gradient instability and network degradation issues, while leveraging attention mechanisms to enhance the mining of critical feature information. Moreover, genomic data processed with frequency encoding was integrated into ReaGP to achieve a richer feature representation.

**Results:**

When assessing the predictive accuracy across three animal datasets and two plant datasets covering 15 traits with varying heritabilities, ReaGP improved predictive performance by 14.41% and 7.78% over linear models specifically genomic best linear unbiased prediction (GBLUP) and BayesB, and by 34.41% and 10.09% over kernel methods namely support vector regression (SVR) and reproducing kernel Hilbert space (RKHS), respectively. ReaGP achieved a 4.35% enhancement on average compared to deep neural network genomic prediction (DNNGP). Furthermore, while ReaGP has more trainable parameters than DNNGP, it requires only half the number of floating-point operations.

**Conclusions:**

We introduced a novel deep learning method for genomic prediction, which integrates residual units, attention mechanisms and frequency-encoded genomic data. Comprehensive evaluation on pig, dairy cow, Huaxi cattle, wheat and rice datasets demonstrated that ReaGP was a promising tool for most traits. Thus, ReaGP could be considered as an efficient deep learning method for genomic prediction in farm animals and crops. The source code in this study is available at https://github.com/LiJing5467/ReaGP.

**Supplementary Information:**

The online version contains supplementary material available at 10.1186/s12711-025-01015-8.

## Background

The advent of genomic selection (GS) signifies the dawn of the whole-genome breeding era, presenting a paradigm shift from the conventional breeding value estimation methods based on phenotypes and pedigrees [1]. GS offers the strategic advantage of early estimation of breeding values in candidate individuals, which is characterized by increased accuracy, and has shorter generational intervals. Such advancements have profound impacts on genetic evaluation systems and schemes for genetic improvement [2–5]. Various methods have been used in GS for animal and plant breeding, such as regression-based models (GBLUP and Bayesian) [6–10]. However, when aiming to capture nonlinear relationships between markers and phenotypes, these models may face two main challenges: (i) the curse of dimensionality, where the number of markers far exceeds that of individuals, leading to overfitting; (ii) an inability to adequately capture non-additive effects among markers.

Machine learning provides effective solutions for dealing with nonlinear problems, and it does not require distributional assumptions for predictive variables [11]. In addition, it rarely requires extensive parameter optimization, as the initial settings often yield satisfactory results, which is also considered as its unique advantage [12–14]. Machine learning methods can be categorized into neural networks, ensemble methods, kernel methods, and other approaches [15]. Kernel methods guide a linear solution in the feature space through a kernel function. Among these, support vector regression (SVR) and reproducing kernel Hilbert space (RKHS) have been widely used in GS. RKHS effectively captures non-additive genetic structures by defining inter-individual covariance through kernel functions and quantifying Euclidean distances via kernel matrices [16]. González-Recio et al. [17] employed four models, including E-BLUP, an F∞-metric model based on linear regression on SNPs (F∞-metric), RKHS, and Bayesian regression (BR), to conduct genomic prediction on mortality rates in a broiler chicken dataset [17]. The results indicated that RKHS achieved favorable prediction accuracy [17]. Other studies have also demonstrated that RKHS exhibited high performance in GS [18–21]. SVR can be interpreted as a type of method based on RKHS, which performs regression on a linear hyperplane in the feature space [22, 23]. Wang et al. [24] demonstrated that SVR outperformed GBLUP by 10% for the T1 trait in a pig dataset and 13.3% for the E1 trait in a wheat dataset.

Deep learning is a type of machine learning technique based on deep and multi-layered neural network architectures, which constructs numerous hidden layers during network training. DL consists of various models, such as deep neural networks (DNNs) [25], and convolutional neural networks (CNNs) [26], among others. DL has demonstrated exceptional capabilities in the genomic prediction of complex traits, owing to its robust self-learning capacity. CNNs have emerged as promising prediction tools, and a variety of deep learning methods based on CNNs have been proposed. For instance, DeepGS showed improvements ranging from 27.70% to 246.34% over DNN, and from 1.44% to 65.24% over RR-BLUP for 8 traits in a wheat dataset [27]. DNNGP achieved 234.2%, 48.9%, and 16.8% higher accuracy than GBLUP, SVR, and DeepGS, respectively, in a wheat dataset. Additionally, ResGS had over 10% higher accuracy than DNNGP, RR-BLUP and three machine learning methods in a maize dataset [28]. SoyDNGP exhibited better predictive performance compared to DNNGP across soybean, maize, rice, potato and cotton datasets [29].

In the field of deep learning, residual networks are among the most successful architectures, using identity mapping to prevent model degradation and compensate for irreversible information loss caused by high non-linearity [30–32]. Attention mechanisms, which are easily integrated with base models such as recurrent networks or CNNs, use back-propagation and offer interpretability to complex neural network models [33]. Some models have incorporated these network components in GS research [28, 29]. Transformers, as an emerging class of models, also utilize attention mechanisms (self-attention and multi-head attention), which play a core role in determining model performance [34]. The combination of ResNet networks and transformers mechanisms in deep learning has achieved promising results across diverse domains [35–37]. However, the combined use of residual units and attention mechanisms is rarely reported in genomic prediction studies. In this study, we integrated residual units with attention mechanisms into CNNs to improve the phenotypic predictive performance and named this proposed framework the residual-attention mechanism for genomic prediction (ReaGP). To better adapt to the CNN framework, we also designed ReaGP with a unique 3D input structure. We evaluated the performance of this new method by comparing it with five popular prediction methods (DNNGP, SVR, GBLUP, RKHS and BayesB) in terms of predictive accuracy across datasets of pig, dairy cow, Huaxi cattle, wheat and rice.

## Methods

### Beef cattle dataset

The Huaxi cattle data were collected from five farms located in Ulgai grassland of the Xilingole League, Inner Mongolia Autonomous Region, China. All animals were born between 2008 and 2022 and were weaned at approximately 6 months of age. After weaning, they were transferred to Jinweifuren Cattle Farm in Beijing for fattening under consistent feeding and management conditions. The growth traits of these animals were measured every six or twelve months until slaughter. Live weight was recorded following a 24-h fasting period. The cattle were slaughtered at ages ranging from 18 to 24 months. Post-slaughter, carcass traits and meat quality traits were measured in accordance with the Institutional Meat Purchase Specifications for Fresh Beef and GB/T 27643–2011. This study measured and analysed three traits: (1) Fattening period daily gain (FDG; kg) calculated as subtracting the live weight upon entry to the farm from the market weight, then dividing by the number of days on the farm. (2) Carcass Weight (CWT; kg) is defined as the body weight post-exsanguination minus the weight of the head, skin, tail, hooves, genital organs and surrounding fat, udder and surrounding fat in cows, and viscera (with kidneys and surrounding fat retained). (3) Weaning Weight (WWT; kg) was adjusted to represent the fasting live weight of calves at 6 months of weaning, using the following equation: (actual weaning weight—birth weight) /180 × age at weaning + birth weight. Huaxi cattle were genotyped using the Illumina BovineHD SNP array. Genotype quality control was performed using PLINK v1.9 software with the following criteria: individuals with missing genotype (> 0.1) were excluded; SNPs were retained with a minor allele frequency (> 0.05), a proportion of missing genotypes (< 0.05), and Hardy–Weinberg equilibrium (p > ${10}^{-6}$). After quality control, the final dataset included 1362 cattle and 574,547 filtered SNPs.

### Public datasets

The pig dataset includes 3,534 animals with five traits from a core farm of the Pig Improvement Company (PIC) [38]. Genotyping was performed using the Illumina PorcineSNP60 chip. After SNP data quality control and phenotype correction, 3,534 individuals with 52,843 SNPs and three selected traits were retained for genomic prediction. The German Holstein cattle dataset consists of 5,024 animals genotyped using the Illumina Bovine SNP50 BeadChip [39, 40]. Three traits with corrected phenotypes for milk yield (MY, kg), milk fat percentage (MFP, %) and somatic cell score (SCS) were used. The number of SNPs after quality control was 42,551. The wheat dataset was retrieved from CIMMYT’s wheat gene bank, containing 2,000 local lines of Iranian bread wheat. Each individual has phenotypes for 8 traits and genotypes for 33,709 SNPs after quality control [41]. The traits of grain length (GL), grain width (GW) and grain protein (GP) were selected for genomic prediction. The rice413 dataset contains 34 traits and 44,100 SNPs [42]. The traits of panicle number per plant (PNPP), seed length (SL) and panicle length (PL) of this dataset were used in our study. The heritabilities of different traits across the five datasets are presented in Table 1.

**Table 1:** The heritabilities of different traits across five datasets

Dataset	Trait	Number of individuals	Heritability
Huaxi cattle	FDG/kg	1299	0.48
	WWT/kg	1362	0.43
	CWT/kg	1303	0.45
Pig	t1	2804	0.07
	t2	2715	0.16
	t5	3184	0.62
Dairy cow	MFP/kg	5024	0.95
	MY/kg	5024	0.94
	SCS	5024	0.88
Wheat	GP	2000	0.86
	GW	2000	0.74
	GL	2000	0.79
Rice	PNPP	372	0.48
	SL	377	0.98
	PL	375	0.66

### Conventional statistical methods

#### GBLUP

1 $$\mathbf{y} = \mathbf{Xb} + \mathbf{Zg} + \mathbf{e},$$

2 $$\mathbf{G} = \frac{{\mathbf{Z}\mathbf{Z}^{\prime } }}{{2\sum\nolimits_{i = 1}^{k} {q_{i} \left( {1 - q_{i} } \right)} }},$$

where, ${\bf \mathrm{y}}$ is an n-dimensional vector consisting of *N* observations, and *N* represents the number of individuals. The matrix **X** is the incidence matrix for the vector ${\bf \mathrm{b}}$ of fixed effects, and **g** is the vector of genomic breeding values associated with the incidence matrix **Z**. The residual vector e is assumed to follow a normal distribution ${\cal N}\left(\mathbf{0},\mathbf{I}{\sigma }_{e}^{2}\right)$, with ${\bf \mathrm{I}}$ being the identity matrix and ${\sigma }_{e}^{2}$ representing the residual variance. Similarly, the genomic breeding values g are assumed to follow a normal distribution ${\cal N}\left(\mathbf{0},\mathbf{G}{\sigma }_{g}^{2}\right)$, where ${\bf \mathrm{G}}$ is the genomic relationship matrix being calculated using Eq. (2) [43], and ${q}_{i}$ is the allele frequency of the *i*^*th*^ marker.

#### BayesB

The BayesB method assumes that only a small portion of markers contribute to genetic effects, while the majority of SNP effects are zero. The proportion of loci with zero effect is denoted as π, while the remaining SNPs follow a normal distribution scaled by a chi-squared distribution. Their effects follow a normal distribution with a mean of zero and a variance of ${\sigma }_{j}^{2}$, and ${\sigma }_{j}^{2}$ follows an inverse chi-squared distribution with degrees of freedom ${v}_{\alpha }$ and a scale factor of ${s}_{\alpha }^{2}$ as shown in Eq. (3) [1],

3 $${\alpha }_{j}|\pi ,{\sigma }_{j}^{2}\sim (idd)\left\{\begin{array}{ll}0 & { \sigma }_{j}^{2}=0 \text{ with probability } \pi, \\{\cal N}(0,{\sigma }_{j}^{2}) & {\sigma }_{j}^{2}>0 \text{ with probability } (1-\pi )\end{array}\right.$$

where $$j=1,...,M$$

$\text{when }{\sigma }_{j}^{2}>\text{0, } { \sigma }_{j}^{2}|{v}_{\alpha },{s}_{\alpha }^{2}{\sim }_{i.d.d.} {v}_{\alpha }{s}_{\alpha }^{2}  \text{ } {\chi}_{{v}_{\alpha }}^{-2},$ where ${v}_{\alpha }$*, *${s}_{\alpha }^{2}$*,* and π are set to 4.2339, 0.0429, and 0.947, respectively. BayesB employs the Metropolis-Hasting algorithm for joint sampling of marker effects and variances.

### Kernel methods

#### RKHS

The RKHS method [44] retains the additive genetic effects under a multivariate normal distribution by integrating the reproducing kernel Hilbert space regression process into the standard linear mixed model. The model is described in Eq. (4),

4 $$\mathbf{y} = \mathbf{W}\mathbf{\theta} + \mathbf{g}\left( \mathbf{x} \right) + \mathbf{e},$$

where ${\bf \mathrm{y}}$ is the vector of phenotypes, ${\bf \mathrm{W}}$ is the incidence matrix for the parametric effects $\mathbf{\uptheta}$ on ${\bf \mathrm{y}}$, and ${\bf \mathrm{e}}$ is the residual vector. $\mathbf{g}(\mathbf{x})$ is a nonparametric function which can be further expressed as Eq. (5),

5 $$\mathbf{g}\left( \mathbf{x} \right) = \mathbf{\alpha_{0}} + \mathop \sum \limits_{i = 1}^{n} \mathbf{\alpha_{i} K}\left( \mathbf{{x - x_{i}} } \right) = \mathbf{\alpha_{0}} + \mathbf{K_{h}{\prime}} \mathbf{\alpha},$$

where $\mathbf{\alpha_{0}}$ is fixed intercept, $\mathbf{\alpha_{i}}$ is a vector of unknown regression coefficients and $\mathbf{K_{h}{\prime}}$ is the kernel matrix generated by a specific smoothing parameter $h$; $\mathbf{K\left( {x - x_{i} } \right)}$ is a reproducing kernel as a basis function. The RKHS, along with the aforementioned GBLUP and BayesB methods, were implemented using the R package “BGLR” [45].

#### SVR

The model of Support Vector Regression (SVR) approach is defined as Eq. (6),

6 $$\mathbf{y} = \mathbf{\beta_{0}} + \mathbf{K}\left( \mathbf{{x,x^{\prime }} } \right) + \mathbf{e},$$

where $\mathbf{\beta_{0}}$ is the intercept term, $\mathbf{K}\left( \mathbf{{x,x^{\prime }} } \right)$ denotes the kernel function that captures the non-linear interactions between observations, and ${\bf {\mathrm{e}}}$ is the residual error. In this study, the radial basis function (RBF) was selected as the kernel function. SVR was implemented in Python version 3.11.0 using “scikit-learn” module [46].

### Deep learning methods

#### DNNGP

The DNNGP model consists of three convolutional layers. Each convolutional layer contains four filters of size 1 × 1 and outputs 64 channels, followed by a Batch Normalization (BN) layer, a Dropout layer, and a Rectified Linear Unit (ReLU) activation function. The implementation of DNNGP source code was provided in Wang et al. [47].

#### ReaGP

**Fig. 1:**
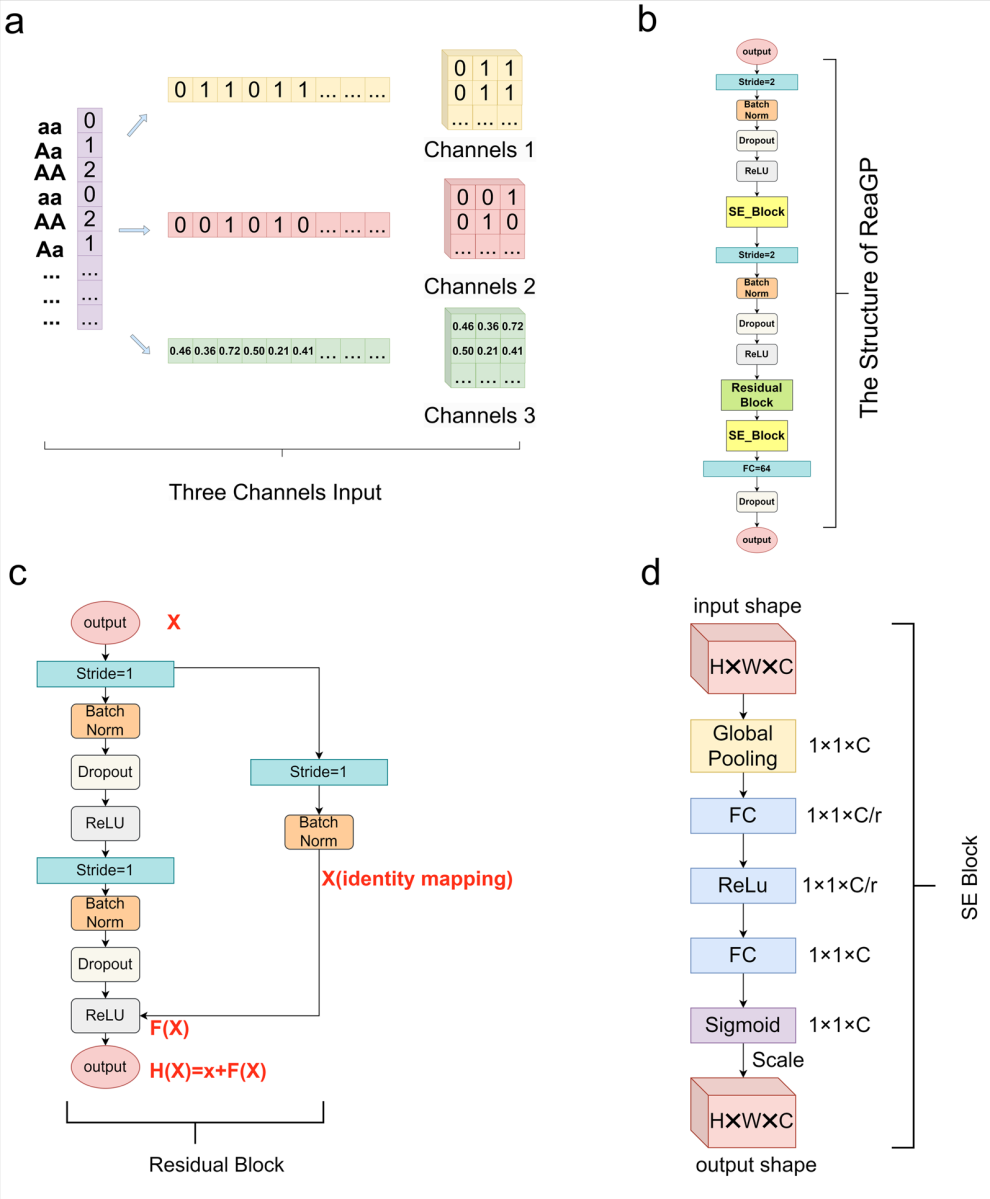
Overview of ReaGP’s input data and the backbone structure. **a** The transformation process of combining genotype data with frequency information as input in ReaGP. **b** Depiction of the ReaGP module structure for regression tasks. **c** Detailed illustration of the residual units. **d** Detailed illustration of a squeeze-and-excitation (SE) block

Inspired by DNNGP, we developed the ReaGP model (Fig. 1). Residual units [48] and attention mechanisms [49] were incorporated into the model architecture to more effectively mitigate the vanishing gradient problem. During the data input phase, data processing libraries from numpy and PyTorch were used to compress genomic data into a 3D matrix with shape (*M*, *M*, 3). Symmetric zero-padding was applied along the width direction when the genome length could not be perfectly square-rooted, ensuring compatibility with the required input tensor shape. The details of the padding are represented in Eq. (7),

7 $$M^2 = \left\{\begin{array}{ll} NN = NumberofSNPs \\ N+P_{L}+P_{R}P_{L},P_{R} = Numberofpadding0, \end{array}\right.$$

For genomic data, traditional categorical encoding represents allelic genotypes as follows: 1 denotes heterozygotes, while 0 and 2 denote homozygotes. In DL, when certain feature values are 0, they contribute little to the final outcome, which might result in the loss of critical information [50, 51]. To address this issue, we adopt a genotype frequency-fused encoding strategy (Encoding 1) in ReaGP, where 0 is represented as (0, 0, freq(aa)), 1 as (1, 0, freq(Aa)), and 2 as (1, 1, freq(AA)). The first two values in each tuple are derived directly from the genotype, while the third value is obtained via frequency encoding based on the Hardy–Weinberg equilibrium formula (Fig. 1a). We also used the one-hot encoding strategy (Encoding 2) [52] as a comparative encoding method in the ablation study.

The ReaGP model comprises of several fundamental modules: the backbone, Residual module, and attention mechanism module. The backbone, as a feature extraction module, includes two convolutional layers, each followed by BN, dropout, and ReLU activation. The first layer uses 64 kernels of size 7 × 7 with a stride of 2 × 2, while the second uses 5 × 5 kernels [53, 54]. Additionally, this module contains two fully-connected layers with 64 and 1 neurons, respectively (Fig. 1b). The residual module employs two identity residual layers. Each residual layer contains two 3 × 3 convolutional layers with a stride of 1 to maintain channel count, followed by BN, dropout, and ReLU layers, using 64 channels. An additional convolutional layer is included to address any mismatch between input and output channels, thereby preventing feature loss (Fig. 1c). Compared with the output $\mathbf{F(x)}$ of the traditional neural network, the output $\mathbf{H(x)}$ of the residual network consists of two parts, $\mathbf{F(x)}$ and identity mapping $x$, as described in Eq. (8),

8 $$\mathbf{H}\left( \mathbf{x} \right) = \mathbf{F}\left( \mathbf{x} \right) + \mathbf{x},$$

During backpropagation, the gradient $\frac{\partial \mathbf{F(x)}}{\partial \mathbf{\theta} }$ can vanish because the network has too many layers, and activation functions (such as $\mathrm{sigmoid}$ or $\mathrm{Tanh}$) cause the gradients to become very small. This issue leads to the vanishing gradient problem ($\frac{\partial L}{\partial \theta }$≈0) as depicted in Eqs. (9) and (10), where L represents the Mean Squared Error (MSE) loss function:

9 $$\frac{\partial L}{{\partial \theta }} = \frac{\partial L}{{\partial H}} \cdot \left( {\frac{\partial F\left( x \right)}{{\partial \theta }} + \frac{\partial x}{{\partial \theta }}} \right),$$

10 $$\mathop {\lim }\limits_{n \to \infty } \frac{\partial F\left( x \right)}{{\partial \theta }} = \frac{{\partial F\left( {L_{n - 1} \left( {L_{n - 2} \left( {...L_{1} \left( x \right)...} \right)} \right)} \right)}}{\partial \theta } = 0,$$

The vanishing gradient problem hinders the normal update of parameters $\theta_{new}$ and affects the learning process, as shown in Eq. (11):

11 $$\theta_{new} = \theta_{old} - \alpha \cdot \frac{\partial L}{{\partial \theta }},$$

However, the identity mapping $x$ in the residual network ensures that the gradient does not vanish, thereby preventing $\frac{\partial L}{\partial \theta }$ from becoming zero and maintaining effective parameter updates. Notably, we introduced squeeze-and-excitation (SE) blocks after the first convolutional layer and residual units to enhance the model’s feature extraction capabilities (Fig. 1d).

### Assessing prediction performance

In this study, tenfold cross-validation (CV) was employed to assess the predictive performance of each method across the five datasets [55–57]. In Scheme I, each dataset was randomly divided into ten groups, where one group without phenotype was used as the test set and the remaining groups used as the training set. For each fold, the current system time was used as the random seed to sample the training set [58]. The predictive accuracy of all methods was compared using the same test set. Predictive accuracy was defined as the average Pearson correlation coefficient values derived from each running. The correlation coefficient was calculated as shown in Eq. (12):

12 $$r\left( \mathbf{y^{*} ,{\mathrm{GEBV}}} \right) = \frac{{{\mathrm{cov}} \left( \mathbf{y^{*} ,{\mathrm{GEBV}}} \right)}}{{\sqrt {{\mathrm{var}} \left( \mathbf{y^{*} } \right){\mathrm{var}} \left( {{\mathbf{GEBV}}} \right)} }},$$

where ${\mathbf{y}}^{*}$ is the corrected phenotype. The tenfold CV was implemented using “Scikit-learn” library [59, 60]. For each method, the CV was repeated 10 times.

To explore the model’s generalization ability, we established Scheme II based on previously reported evaluation criteria [61]. In Scheme II, each dataset was divided into 3 subsets, 10% formed the test subset, 10% formed the validation subset, and the remaining 80% formed the training subset. The detailed workflow of Scheme II was as follows: (1) reshuffling validation-training split within the 90% non-test data, (2) training models on the newly defined 80% training subset, (3) selecting optimal hyperparameters via the 10% validation subset, and (4) evaluating the final performance of the optimized model on the fixed 10% test set. Final evaluation metrics were computed as the average of results across 10 independent evaluations (see Additional file 1, Fig. S1) [62, 63].

To evaluate the complexity of deep learning models, we employed Floating Point Operations (FLOPs) and Parameters (Param) as metrics in Scheme II [29], specifically. 1 M FLOPs means that one million floating-point operations are involved in a single inference or computation with the model.1 M Param indicates that the model contains one million trainable parameters. Additionally, we assessed the phenotypic prediction performance of deep learning models via scheme II [28]. This assessment included the regression coefficient between the observed and predicted phenotypes, and the distribution pattern of predicted versus observed phenotypes.

### Computing environment

Computation was performed on a Precision 7920 Tower Server equipped with two 24-core Intel® Xeon® Gold 6248R processors (3.00 GHz), 944 GB of main memory and RTX A6000 GPUs. The DNNGP and ReaGP models were trained on different dataset using the Adam and SGD optimizers with a weight decay of 10^–5^. The epoch that achieved the best performance on the test set was preserved as the final model. The ReaGP model was implemented using PyTorch (version 2.2.1). The hyperparameter configurations for all methods are provided (see Additional file 2, Table S1).

## Results

### Prediction accuracies of six models under schemes I

**Fig. 2:**
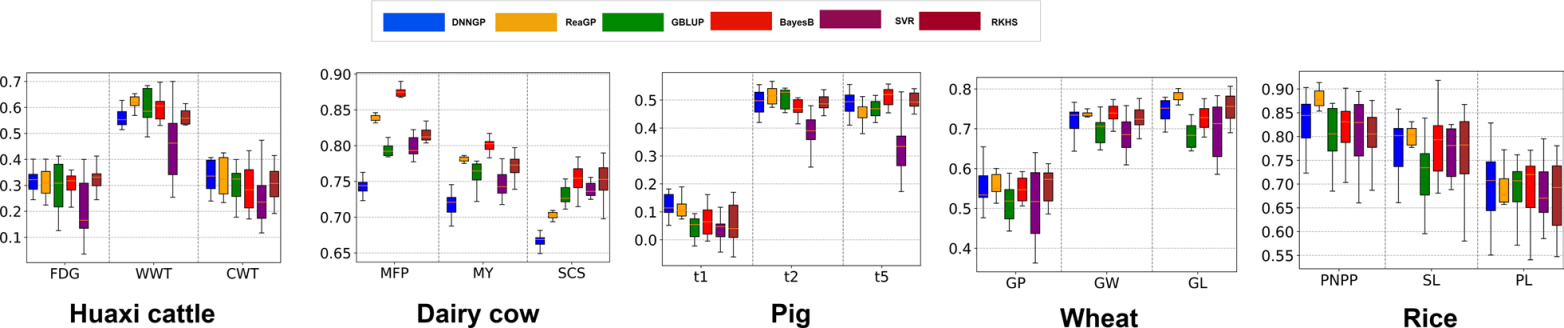
Predictive accuracies of different models across 15 traits

We evaluated the predictive performance of six models on the Huaxi cattle, dairy cow, pig, wheat and rice datasets (Fig. 2). In animal datasets, the average prediction accuracies of DNNGP for the three traits in Huaxi cattle were 0.308 (FDG), 0.551 (WWT), and 0.334 (CWT). The average prediction accuracies of ReaGP were 0.313 (FDG), 0.615 (WWT), and 0.333 (CWT). We found that ReaGP emerged as the optimal model for genomic prediction, outperforming DNNGP, BayesB, GBLUP, SVR, and RKHS in all three traits by an average of 4.31%, 8.34%, 6.78%, 40.75% and 6.25%, respectively, in terms of the prediction accuracies (see Additional file 3, Table S2). SVR exhibited the lowest accuracy among all models for the three traits. For FDG and CWT, two neural network models (DNNGP and ReaGP) achieved higher accuracies than the other four models. For the dairy cow dataset, BayesB achieved the highest predictive performance across all traits. It is worth noting that RKHS and BayesB yielded similar accuracies in the SCS trait (0.754 and 0.751, respectively) (see Additional file 4, Table S3). Among the two neural network models (DNNGP and ReaGP), ReaGP ranked second in predictive accuracy for MY and MFP. In contrast, DNNGP was obviously inferior to ReaGP as well as the other four models. For the pig dataset, the overall predictive accuracy was relatively low. For the t1 trait, the prediction accuracies of GBLUP, BayesB, SVR, RKHS, DNNGP, and ReaGP were 0.054, 0.069, 0.035, 0.060, 0.124 and 0.121, respectively. For ReaGP, the average prediction accuracies for t1, t2 and t5 were 0.121, 0.516, and 0.468; for DNNGP, the values were 0.124, 0.494, and 0.489. Compared with BayesB, GBLUP, SVR and RKHS, the average prediction accuracy of t1, t2 and t5 traits of ReaGP were 4.6%, 8.3%, 46.3% and 5.4% higher, respectively (see Additional file 5, Table S4).

In the crop dataset, the average prediction accuracies of ReaGP in the wheat dataset for GP and GL traits were 0.622 and 0.783, respectively, outperforming DNNGP, GBLUP, BayesB, SVR and RKHS for these two traits by an average of 8.9%, 18.6%, 10.4%, 17.2% and 7.7% in prediction accuracy. For GW, BayesB achieved the highest accuracy (0.740), while ReaGP exhibited nearly equivalent performance (0.738) (see Additional file 6, Table S5). DNNGP did not achieve higher accuracy than RKHS, BayesB and SVR. The rice dataset exhibited very high accuracies overall with all six methods achieving accuracies exceeding 0.8. During the tenfold cross-validation, DNNGP and ReaGP even reached accuracies as high as 0.9 in several replicates. Among all models, ReaGP achieved the highest average prediction accuracy (0.881) and outperformed DNNGP by 7.2% and 1.4% for PNPP and SL, respectively. For PL, however, DNNGP achieved a slightly higher accuracy (0.697) than ReaGP (0.694) (see Additional file 7, Table S6).

### Comparison of generalization ability and complexity between ReaGP and DNNGP

**Fig. 3:**
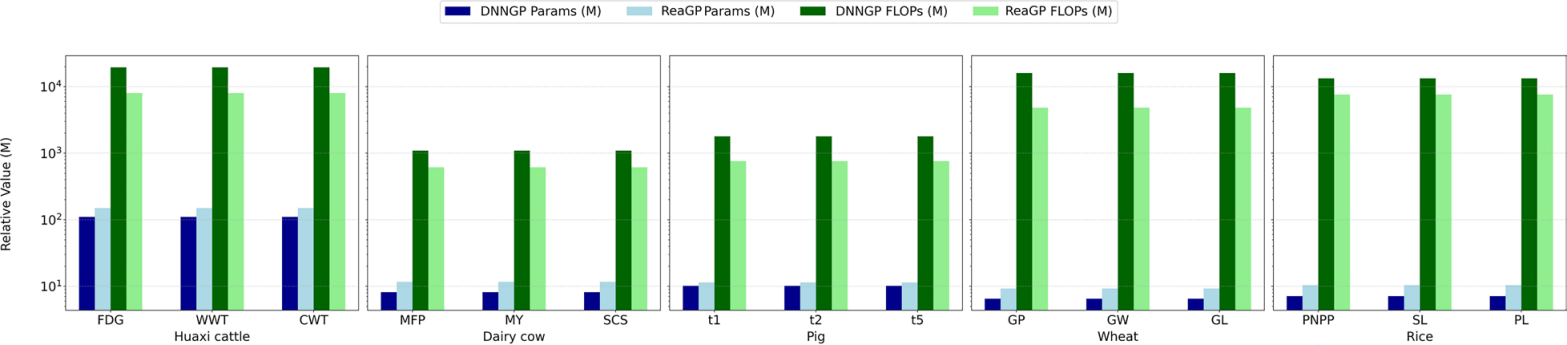
Comparison of predictive accuracies between ReaGP and DNNGP under scheme II across five datasets

To further evaluate the generalization ability of the two deep learning models, we utilized scheme II as the evaluation criterion to assess predictive accuracy (Fig. 3). Overall, ReaGP exhibited superior accuracy and more robust generalization ability compared with DNNGP across all traits in the five datasets, except for FDG (Huaxi cattle) and t2 (pig) traits (see Additional file 8, Table S7). Figure 3 also illustrated the difference in predictive accuracies between scheme I and scheme II. The accuracy of scheme II was slightly lower than that of scheme I across all datasets and traits, except for t2 (pig) and GW (wheat) traits.

For the Huaxi cattle dataset, prediction accuracies of ReaGP for WWT and CWT were 8.74% and 83.3% higher than those of DNNGP, respectively. For FDG, the predictive accuracies of DNNGP and ReaGP were 0.074 and 0.073 (negligible difference), respectively. The average accuracy of the three traits under scheme II was 23.5% lower than that under scheme I. For the dairy cow dataset, ReaGP’s predictive accuracies for the three traits (MY, MFP, SCS) were 12.3%, 8.91% and 4.82% higher than those of DNNGP, respectively. The average accuracy of three traits under scheme II was 2.54% lower than that under scheme I. In the pig dataset, ReaGP outperformed DNNGP in t1 and t5 by 39.92% and 0.24%, respectively. Additionally, the predictive accuracy of t2 was nearly identical between scheme I and scheme II. In the wheat dataset, the average prediction accuracies of scheme II for GP and GL were 18.5% and 14.6% lower than those of scheme I, respectively. For GW, the predictive accuracy under scheme I was similar to that under scheme II. For the rice dataset, both deep learning models achieved lower average predictive accuracies under scheme II than those under scheme I for three traits (PNPP, SL and PL), with decreases of 6.5%, 3.1% and 5.3%, respectively. Notably, under scheme II, ReaGP’s predictive accuracy for SL was 13.8% higher than that of DNNGP, and 1.4% higher than its own accuracy under scheme I.

**Fig. 4:**
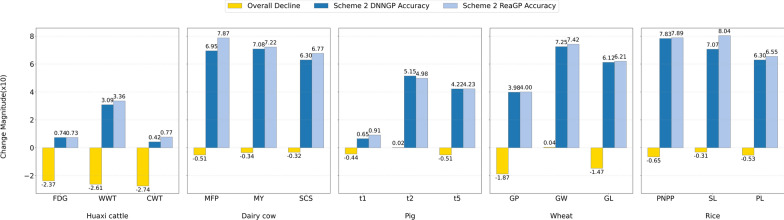
Comparison of Params and FLOPs between ReaGP and DNNGP across five datasets

To compare the complexity of two deep learning models, we calculated the Params and FLOPs for DNNGP and ReaGP. Even though ReaGP had more parameters than DNNGP, it required only half the number of floating-point operations compared with DNNGP across all traits in the five datasets (Fig. 4).

### Phenotypic prediction performance of ReaGP under scheme II

**Fig. 5:**
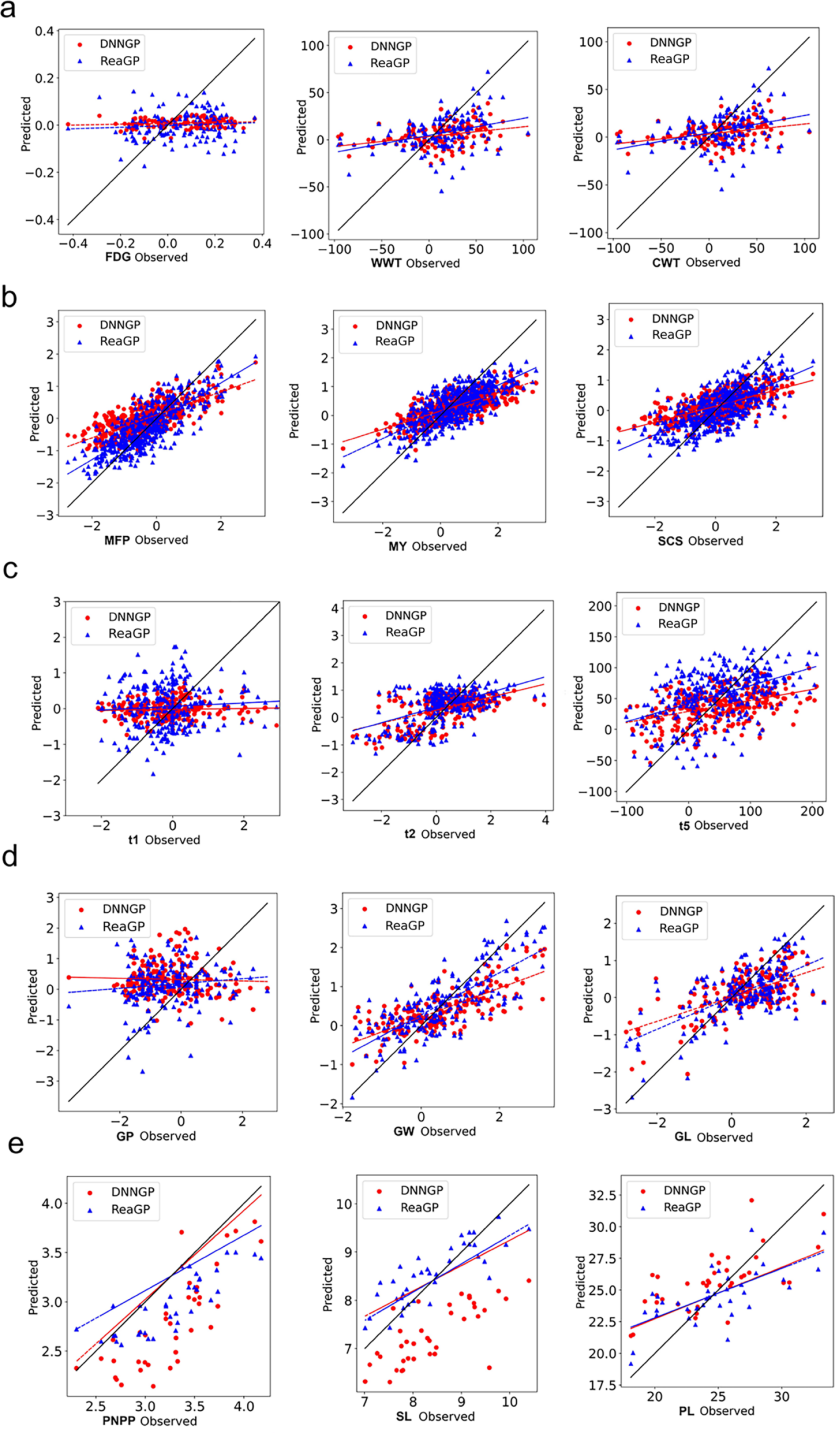
Comparison of fitted curves for observed values, DNNGP predicted values, and ReaGP predicted values across five datasets From left to right: **a** Huaxi cattle (FDG, WWT, CWT), **b** dairy cow (MFP, MY, SCS), **c** pig (t1, t2, t5), **d** wheat (GP, GW, GL), **e** rice (PNPP, SL, PL).

**Fig. 6:**
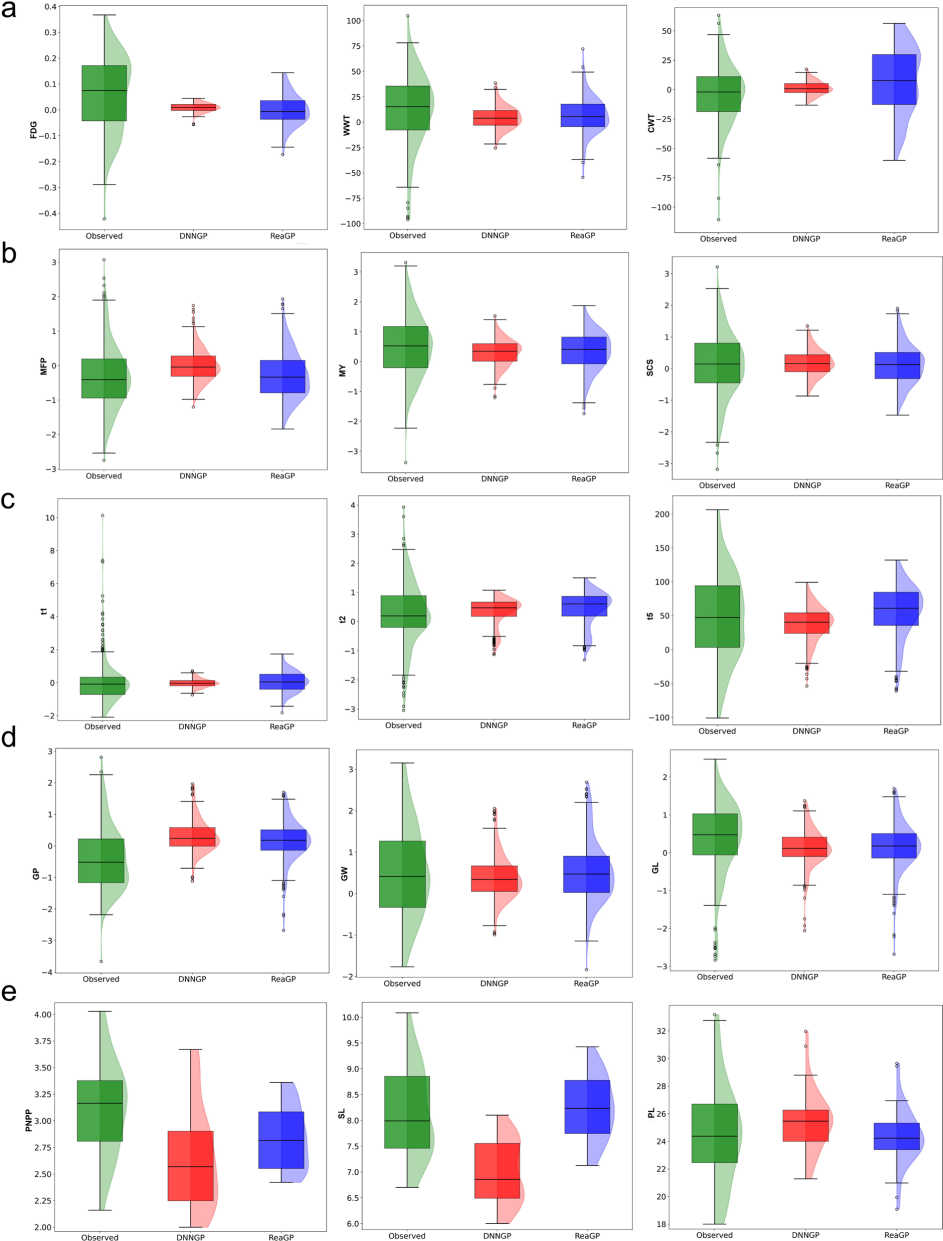
Comparison of the distributions of observed and predicted phenotypes for ReaGP and DNNGP across five datasets From left to right: **a** Huaxi cattle (FDG, WWT, CWT), **b** dairy cow (MFP, MY, SCS), **c** pig (t1, t2, t5), **d** wheat (GP, GW, GL), **e** rice (PNPP, SL, PL).

Given that the differences in the generalization ability between ReaGP and DNNGP were more pronounced under Scheme II, we further investigated their phenotypic prediction performance using Scheme II results (Fig. 5). To compare the predictive capabilities of the two models more comprehensively, we analyzed their prediction distributions across all traits (Fig. 6). In animal datasets, we found deep learning methods’ predictive ability was clearly displayed in MFP, MY and SCS, especially for MFP in dairy cow dataset. As shown in Fig. 5b, ReaGP exhibited a steeper fitting line slope, compared with DNNGP, which indicated that the range of phenotypes predicted by ReaGP was more extensive. In Fig. 6b, the MFP phenotype followed a normal distribution, with minimum, 25th percentile, median, 75th percentile, and maximum values of  −3.0,  −0.2, 0.9, and 3.9, respectively. The range of predicted phenotypes for DNNGP was between  −1.1 and 1.1, while for ReaGP, the range was between  −1.3 and 1.5. Both predicted ranges were narrower than the observed range.

In the rice and wheat datasets, ReaGP achieved higher accuracies for PNPP and PL traits, however, for PNPP, DNNGP predicted a wider range of phenotypes. Statistical analysis revealed similar predictive power between DNNGP (*y* = 0.408*x* + 14.570) and ReaGP (*y* = 0.390*x* + 15.018) for PL (Fig. 5e). In Fig. 6e, DNNGP produced broader prediction distributions for PNPP and SL than ReaGP. Notably, DNNGP’s predicted phenotypes for PNPP and SL exceeded the range of observed phenotype.

### Ablation study

**Fig. 7:**
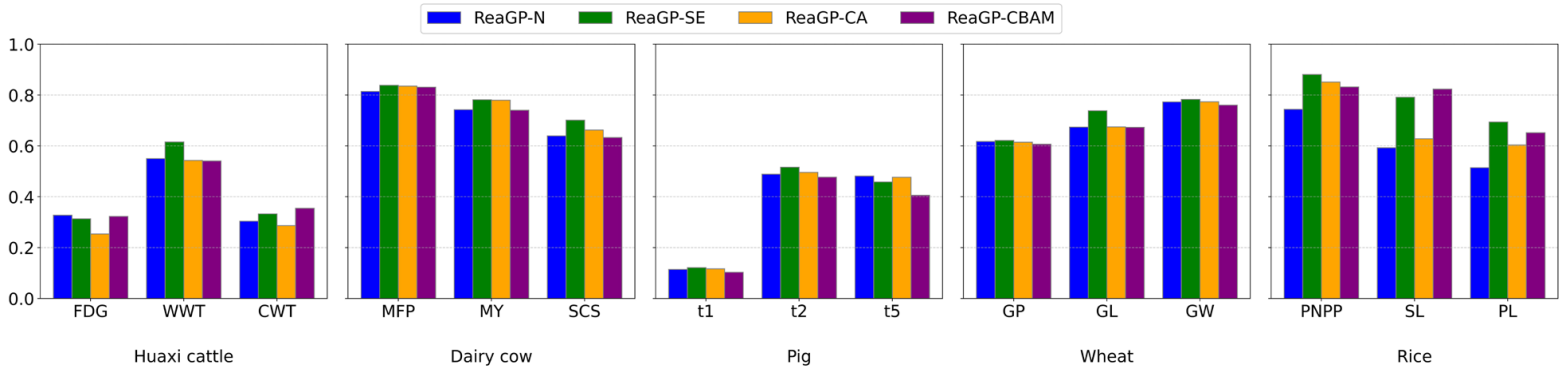
Predictive accuracies of different attention mechanisms across five datasets

To investigate the impact of attention mechanisms on genomic prediction accuracy, we evaluated the performance of four variants of ReaGP model. ReaGP-N, ReaGP-SE, ReaGP-CA, and ReaGP-CBAM, respectively refer to baseline network structures that incorporate no attention mechanisms, the squeeze-and-excitation (SE) attention mechanism, coordinate attention (CA), and the convolutional block attention module (CBAM). We found that the average predictive accuracy across traits of ReaGP was 8.8%, 4.2% and 5.9% higher than that of ReaGP-N, ReaGP-CBAM and ReaGP-CA, respectively (Fig. 7 and see Additional file 9, Table S8). Moreover, in the dairy cow and Huaxi cattle datasets, accuracies of ReaGP-CBAM and ReaGP-CA were lower than those of ReaGP-N. The results indicated that the SE attention mechanism in ReaGP can capture more intrinsic nonlinear genomic features compared with CBAM and CA.

We evaluated the ReaGP model’s performance using two different genotype encoding strategies, Encoding 1 (genotype frequency-fusion encoding) and Encoding 2 (one-hot encoding). The prediction accuracies of the two encoding strategies were visualized in radar charts (see Additional file 10, Fig. S2). In these charts, higher predictive accuracies are presented by positions in the outer ring, while lower accuracies correspond to the inner ring. As observed from the figure, Encoding 1 outperformed Encoding 2 for traits SL, PL, SCS and WWT. In contrast, Encoding 2 outperformed Encoding 1 only for the traits t1 and MY. In terms of MSE, Encoding 1 yielded lower values than Encoding 2. Overall, Encoding 1 exhibited superior performance to Encoding 2 in both prediction accuracy and MSE (see Additional file 11, Fig. S3).

## Discussion

### Synthesizing deep learning techniques for enhancing ReaGP’s predictive accuracy

GS serves as a powerful tool in animal and crop breeding, offering high predictive accuracy and shortening generational intervals. Linear regression and conventional machine learning methods might not provide satisfactory predictive accuracy when dealing with complex traits. Although some deep learning approaches have achieved better outcomes in GS, they still have some limitations. In image analysis, max pooling is widely adopted in CNN architectures for images to eliminate the effects of image jitter and improve predictive performance [64]. In GS, deep learning models such as DeepGS and DNNGP, which incorporate max pooling layers, can effectively capture the critical SNP features. However, they may overlook minor features, potentially leading to suboptimal predictive accuracy [28]. Inspired by ResGS and SoyDNGP, we adopt stride convolution (with a stride of 2) instead of pooling layers for information compression, thereby retaining essential features.

In the input layer of deep learning models like DeepGS and DNNGP, data were organized in the form of one-dimensional vector [27, 47], which limited their capacity to capture genotypic variation information. In contrast, we employed three-dimensional vector data in the input layer of ReaGP to increase the parameter volume, which can enhance the model’s capacity to extract complex feature information [29]. Additionally, the convolution kernel size in DNNGP and DeepGS was set to 4 [27, 47], which restricted the models’ feature extraction capability. We replaced this fixed kernel size with a larger convolution kernel, which expands the receptive field and thus enables the capture of more global information [53, 54].

Notably, a larger convolution kernel and the integration of attention mechanisms and residual block can increase FLOPs, which means the model requires more computational resources [65]. However, in this study, we reduced FLOPs to fewer than half of those required by DNNGP through the use of stride convolution. We also adopted the training strategies from existing deep learning models, incorporating Dropout and Batch Normalization to prevent overfitting, applying L1 regularization to reduce model complexity, and using early stopping strategies to enhance training efficiency [28, 47].

### Attention mechanism and frequency information play an important role in genomic prediction

Attention mechanisms can enhance the model’s predictive performance due to their ability to allocate distinct weights to different parts of the input, enabling the model to focus on processing the most relevant information [49, 66–68]. We adopted SE, CBAM, and CA as benchmarks in our study, because they represent distinct approaches (channel, channel + spatial, and long-range spatial attention) and are widely used in deep learning [29, 69, 70]. In our study, the model incorporating attention mechanisms outperformed the model without attention mechanism for 15 traits across five datasets. The predictive accuracies of ReaGP incorporating SE were higher across five datasets than those of ReaGP with other attention mechanisms. Unlike CBAM and CA, the SE mechanism concentrated on the channel dimension, employing global average pooling to extract spatial features and using fully connected layers to learn the weights for each channel [71]. Furthermore, the SE structure was notably simpler, reducing the computational complexity for specific tasks within limited resources [72]. The computational efficiency and channel-specific focus of the SE mechanism rendered it exceptionally effective in ReaGP [71].

Regarding encoding strategies, incorporating frequency information into ReaGP captured crucial features and increased predictive accuracy, which was consistent with Jubair [73]. In genomic selection, allele frequencies can significantly impact the predictive accuracy [74]. For instance, the MAF of QTL influenced the predictive ability of quantitative traits in single-breed and multi-breed populations [75–78]. In our study, we incorporated allele frequency information into the encoding process of three channels to capture more critical features. This approach enhanced predictive accuracy and reduced the MSE, demonstrating the potential of exploiting frequency information to improve genomic prediction performance.

### Comprehensive performance of ReaGP

In terms of predictive accuracy, for certain traits ReaGP considerably outperformed the other methods in this study. In GS, models are typically trained using the complex relationships between SNPs and phenotypes. Different models exhibited varying performance across diverse genetic architectures of traits in the five datasets. For linear methods, BayesB achieved higher prediction accuracies than the other five models for three traits in the dairy cow dataset (Fig. 2). The predictive performance of BayesB was consistent with previous published studies [79–81]. This can be attributed to the Bayesian methods’ assumptions, which may be better suited for fitting the genetic architecture consisting of a few loci with large effects on quantitative traits [82]. For deep learning, in most cases, ReaGP achieved higher prediction performance compared with DNNGP. We also found that ReaGP performed better than DNNGP for most traits in plant datasets and a few traits in animal datasets, which indicated incorporating the residual units and attention mechanisms might be an efficient strategy in deep learning. For traits with high heritability (e.g., those in the plant datasets and the dairy cow dataset), ReaGP achieved higher accuracy than that of DNNGP. Moreover, for traits with low and medium heritability (t2 trait in pigs, FDG and WWT traits in Huaxi cattle), the accuracy of ReaGP was superior to that of DNNGP. After evaluating different genomic prediction approaches across traits with varying heritabilities, these findings suggest that ReaGP is better suited to the genetic architectures of complex traits. Notably, even in small sample sizes (413 individuals in rice dataset), ReaGP still attained high predictive performance across traits with different genetic architectures.

From a generalization ability perspective, accuracies under scheme II were slightly lower than those under scheme I. This is consistent with Berlin’s theory, which primarily attributes this difference to the use of the validation set for optimal model selection and the prohibition against further model adjustments after the final model assessment with the test dataset. The model has, to some extent, “adapted” to the validation set, resulting in better outcomes on the validation set, while the accuracy may be lower on the test dataset [83]. Nevertheless, appropriate methods still demonstrated robust performance on the test set. While DNNGP was superior for the FDG and t2 traits, but ReaGP performed better in genomic prediction for more traits. For FLOPs, although its unique model structure leads to a theoretical reduction in FLOPs, implementation still requires support from the underlying hardware [84]. We found that ReaGP consumed fewer computational resources than DNNGP in the same GPU environment. This improvement can be attributed not only to the architectural design of the backbone network but also to the incorporation of residual units, which effectively reduce the computational complexity of the model [48, 85]. In addition, residual units can solve the degradation problem by adopting shortcut connections to balance nonlinear learning and identity transformation [86]. By incorporating the residual units, ReaGP ensures gradient flow and training stability in ultra-deep neural networks [87].

For complexity and interpretability, the integration of the residual units and attention mechanisms in deep learning has significantly advanced image processing tasks. Residual units ensure effective gradient propagation, while attention mechanisms enable focused extraction of key features. This combined approach prevents gradient vanishing while achieving more precise feature extraction. Inspired by structural similarities between imaging and genomic data: such as (i) high-dimensional features (pixels vs. high-density SNP markers) [88, 89], (ii) local correlations (edge textures vs. linkage disequilibrium, LD) [90, 91], and (iii) sparse signals (small target regions vs. oligogenic architectures with major/minor QTLs) [92, 93], we adapted the residual units and attention mechanisms to genomic selection. Our approach demonstrated notable improvements in prediction accuracy, though its biological interpretation remains unexplored.

## Conclusions

ReaGP, a novel deep learning genomic prediction framework incorporating residual units, attention mechanisms and frequency information, was proposed to improve the predictive accuracy. Comparative genomic prediction analyses were conducted between ReaGP and six reference method (BayesB, SVR, RKHS, DNNGP, and GBLUP) across pigs, dairy cow, Huaxi cattle, wheat, and rice dataset. The results demonstrate that ReaGP outperformed other methods for most traits in the two plant datasets and a few traits in the three animal datasets. For future work, the integration of other mechanisms into the model could be explored to further evaluate ReaGP’s performance.

## Supplementary Information

Supplementary Material 1

Supplementary Material 2

Supplementary Material 3

Supplementary Material 4

Supplementary Material 5

Supplementary Material 6

Supplementary Material 7

Supplementary Material 8

Supplementary Material 9

Supplementary Material 10

Supplementary Material 11

## Data Availability

Huaxi dataset: 10.5061/dryad.4qc06. Dairy cow dataset: https://www.g3journal.org/content/5/4/615.supplemental. Pig dataset: http://www.g3journal.org/lookup/suppl/doi:10. 1534/g3.111.001453/-/DC1. Wheat dataset: http://genomics.cimmyt.org/mexican_iranian/traverse/iranian/standarizedData_univariate.RData. Rice dataset: http://www.ricediversity.org/44kgwas.
